# *SEQUENCE SLIDER*: expanding polyalanine fragments for phasing with multiple side-chain hypotheses

**DOI:** 10.1107/S2059798320000339

**Published:** 2020-02-25

**Authors:** Rafael Junqueira Borges, Kathrin Meindl, Josep Triviño, Massimo Sammito, Ana Medina, Claudia Millán, Martin Alcorlo, Juan A. Hermoso, Marcos Roberto de Mattos Fontes, Isabel Usón

**Affiliations:** a Crystallographic Methods, Institute of Molecular Biology of Barcelona (IBMB–CSIC), Baldiri Reixach 15, 08028 Barcelona, Spain; b Departamento de Física e Biofísica, Instituto de Biociências, Universidade Estadual Paulista (UNESP), Botucatu-SP 18618-689, Brazil; c Department of Haematology, Cambridge Institute for Medical Research, University of Cambridge, Hills Road, Cambridge CB2 0XY, England; d Department of Crystallography and Structural Biology, Instituto de Química-Física ‘Rocasolano’, Consejo Superior de Investigaciones Científicas (CSIC), 28006 Madrid, Spain; e ICREA at IBMB–CSIC, Baldiri Reixach 13-15, 08028 Barcelona, Spain

**Keywords:** phasing, *SEQUENCE SLIDER*, molecular replacement, fragment-based molecular replacement, side-chain extension, *ARCIMBOLDO*, *Phaser*, *SHELXE*

## Abstract

Fragment-based molecular-replacement methods can solve a macromolecular structure quasi-*ab initio*. *ARCIMBOLDO*, using a common secondary-structure or tertiary-structure template or a library of folds, locates these with *Phaser* and reveals the rest of the structure by density modification and autotracing in *SHELXE*. The latter stage is challenging when dealing with diffraction data at lower resolution, low solvent content, high β-sheet composition or situations in which the initial fragments represent a low fraction of the total scattering or where their accuracy is low. *SEQUENCE SLIDER* aims to overcome these complications by extending the initial polyalanine fragment with side chains in a multisolution framework. Its use is illustrated on test cases and previously unknown structures. The selection and order of fragments to be extended follows the decrease in log-likelihood gain (LLG) calculated with *Phaser* upon the omission of each single fragment. When the starting substructure is derived from a remote homolog, sequence assignment to fragments is restricted by the original alignment. Otherwise, the secondary-structure prediction is matched to that found in fragments and traces. Sequence hypotheses are trialled in a brute-force approach through side-chain building and refinement. Scoring the refined models through their LLG in *Phaser* may allow discrimination of the correct sequence or filter the best partial structures for further density modification and autotracing. The default limits for the number of models to pursue are hardware dependent. In its most economic implementation, suitable for a single laptop, the main-chain trace is extended as polyserine rather than trialling models with different sequence assignments, which requires a grid or multicore machine. *SEQUENCE SLIDER* has been instrumental in solving two novel structures: that of MltC from 2.7 Å resolution data and that of a pneumococcal lipoprotein with 638 residues and 35% solvent content.

## Abstract 2

When phasing cannot be accomplished from a partial polyalanine starting model, extending the model with side chains in a multi-solution way may succeed. *SEQUENCE SLIDER* implements this approach for use in *ARCIMBOLDO*.

## 1. Introduction   

Molecular replacement (MR) is nowadays the most prevalent method of addressing the crystallographic ‘phase problem’ by approximating the phases with those derived from a homologous protein of known structure placed into the target unit cell (Rossmann & Blow, 1962 ▸). The implementation of more sensitive and accurate maximum-likelihood targets in MR (Read, 2001 ▸) allowed the advent of fragment-based methods, which are between *ab initio* phasing (Usón & Sheldrick, 1999 ▸) and MR. Common secondary-structure or tertiary-structure fragments are used; thus, no specific structural knowledge of the target structure is required, but MR methods are needed for correct placement. It is then necessary to extend from the partial structure composed of the fragments to a fairly complete and thus interpretable structure. Early methods explored the use of model α-helices (Glykos & Kokkinidis, 2003 ▸; Rodríguez *et al.*, 2009 ▸) and RNA secondary-structure elements, combining manual map inspection, refinement, density modification and composite OMIT maps (Robertson & Scott, 2008 ▸; Robertson *et al.*, 2010 ▸). Currently, a number of pipelines implement fragment-based phasing, relying on the rotation (Storoni *et al.*, 2004 ▸) and translation (McCoy *et al.*, 2005 ▸) functions in *Phaser* to locate small, yet very accurate fragments. Sometimes, even if *Phaser* produces correct solutions, distinguishing them among many false solutions may not be possible as the expected log-likelihood gain (eLLG) that they would be expected to render if correctly placed is inconclusive. For small search models, correct and incorrect solutions are frequently characterized by similar figures of merit. Thus, many hypotheses are pursued in parallel and success in extending some of them into a full solution serves to identify the correct solutions.

The phases from the partial solutions need to be further improved using sophisticated techniques: extrapolation of nonmeasured data (Usón *et al.*, 2007 ▸; Caliandro *et al.*, 2005*a* ▸,*b* ▸; Dodson & Woolfson, 2009 ▸), density modification using the *VLD* algorithm (Caliandro *et al.*, 2014 ▸), hybrid Fourier syntheses (Burla *et al.*, 2011 ▸) and charge flipping (Palatinus, 2013 ▸). The iterative process of phase and map improvement with map interpretation in a protein trace may reveal the rest of the structure for the true solution. Frequently, *SHELXE* (Thorn & Sheldrick, 2013 ▸) is used to improve the phases, but *ACORN* (Foadi, 2003 ▸), *RESOLVE* (Terwilliger, 2004 ▸) and *Buccaneer* (Cowtan, 2006 ▸) implement alternative methods. A prominent route for the extension of partial solutions all the way to practically complete model building is provided by *ARP*/*wARP* (Perrakis *et al.*, 2001 ▸; Chojnowski *et al.*, 2020 ▸), which alternates density modification and model building with refinement with *REFMAC*5 (Murshudov *et al.*, 2011 ▸).

*ARCIMBOLDO* (Millán *et al.*, 2015 ▸) exploits common small secondary-structure or tertiary-structure fragments. It combines MR with *Phaser* (McCoy *et al.*, 2007 ▸) with density modification and autotracing with *SHELXE* (Sheldrick, 2010 ▸). The original algorithm has been extended to incorporate other sources of information (Rodríguez *et al.*, 2012 ▸) and diversified to use libraries of fragments (Sammito *et al.*, 2013 ▸). Suitable fragments from distant homologs can also be identified (Sammito *et al.*, 2014 ▸) or improved (Millán *et al.*, 2018 ▸), decomposing templates into fragments to refine subsequent degrees of freedom (McCoy *et al.*, 2018 ▸). Decisions are guided by the eLLG (Oeffner *et al.*, 2018 ▸). Fragment-based phasing methods are becoming very popular in successful pipelines such as *AMPLE* (Bibby *et al.*, 2012 ▸; Rigden *et al.*, 2018 ▸; Keegan *et al.*, 2015 ▸; Simpkin *et al.*, 2019 ▸), which uses *Rosetta* (Qian *et al.*, 2007 ▸), *QUARK* (Xu & Zhang, 2012 ▸) or *CONCOORD* (de Groot *et al.*, 1997 ▸) to generate models, offering the possibility of generating *ab initio* fragments derived from the target sequence, *Fragon* (Jenkins, 2018 ▸) or *FRAP* (Shrestha & Zhang, 2015 ▸).

Fragment placement is becoming increasingly successful owing to a number of advances in accounting for errors, conditioning the problem and scoring the solutions. The new intensity-based targets accounting for measurement and model coordinate errors in *Phaser* (Read & McCoy, 2016 ▸), refinement of the model root-mean-square deviation (r.m.s.d.) and model improvement refining internal degrees of freedom or through eLLG pruning bring about cases in which a correct but partial solution can be confidently identified, whereas its extension through density modification and autotracing still fails. Such situations are related to a resolution of the diffraction data of worse than 2.0 Å, a solvent content below 40%, predominant β-sheet composition, situations in which the initial fragments represent only a low fraction of the total structure and error in accuracy of the placed fragment. One possible way to overcome the named complications would be to extend the initial polyalanine fragment with side chains. The incorporation of side chains based on real-space electron density and on known sequence is applied in model-building algorithms such as *ARP*/*wARP* (Langer *et al.*, 2008 ▸), *RESOLVE* (Terwilliger, 2004 ▸) and *Buccaneer* (Cowtan, 2006 ▸). In the case of *ARCIMBOLDO*, we target the scenario in which the maps generated from small fragments or very partial solutions do not yet render details for side-chain discrimination. Therefore, multiple hypotheses need to be generated and explored, subject to the restrictions imposed by the known sequence. Again, multiple hypotheses with different sequence assignments need to be evaluated and trialled through expansion, where an improvement in the trace and the correlation coefficient (CC) calculated from the traced atoms (Fujinaga & Read, 1987 ▸) will indicate structure solution. In an iterative way, this method allows the automatic solution and building of macromolecules. The solution using *ARCIMBOLDO* of the previously unknown structure of MltC at 2.7 Å resolution provided proof of concept (Artola-Recolons *et al.*, 2014 ▸) and the present implementation was instrumental in solving the novel structure of a lipoprotein containing 659 amino acids. Here, we describe the implementation of a multisolution method of sequence extension which provides two different modes depending on the starting substructure: in the case of search fragments derived from a remote homolog in *ARCIMBOLDO_SHREDDER* or a partial MR solution the alignment between the sequence of this structure and the target provides a restriction, whereas for disconnected traces or general fragments secondary-structure prediction from the target sequence is the only previous information and many more possibilities need to be considered.

## 2. Materials and methods   

### 2.1. Computing settings   

Structure solution and tests were run on a local HTCondor version 8.4.5 (Tannenbaum *et al.*, 2003 ▸) grid made up of 160 nodes totalling 225 Gflops. The submitter machine was a six-core workstation with 24 GB RAM running Ubuntu Linux. Running times on the grid depend on the size of the atomic model and the number of evaluated hypotheses; the cases described in this paper typically took from 30 min to 20 h.

### 2.2. Software versions   

The experiments described in this study were run with *SEQUENCE SLIDER*, which is developed in Python (version 2.7, compatible with Python3). It is distributed together with *ARCIMBOLDO* through *CCP*4 (Winn *et al.*, 2011 ▸). It uses the following external crystallographic programs. Side chains are generated with *SCWRL *(version 4.0; Krivov *et al.*, 2009 ▸), and partial models are refined with either *BUSTER* (version 2.10.3; Bricogne *et al.*, 2018 ▸), *REFMAC*5 (version 5.8.0238; Murshudov *et al.*, 2011 ▸) or *phenix.refine* (version dev_3405; Afonine *et al.*, 2012 ▸) and expanded with *SHELXE* (version 2011 for the original solution of MltC and version 2019/1 for all tests; Usón & Sheldrick, 2018 ▸). The figure of merit used in decision making is the intensity-based log-likelihood gain (LLG) calculated by *Phaser* (version 2.8.2), fixing the r.m.s.d. after its optimization on the initial model (Oeffner *et al.*, 2013 ▸; Read & McCoy, 2016 ▸). The correctness of the *SEQUENCE SLIDER* models from our tests is assessed at different stages. Structure-amplitude-weighted mean phase errors (wMPEs; Lunin & Woolfson, 1993 ▸) of models before and after refinement and from traces are calculated by *SHELXE* against the final deposited models. The correctness of hypotheses is calculated by *SEQUENCE SLIDER* using the identity between pairs of C^α^ atoms in the final and partial models within a maximum radius of 1.0 Å. The r.m.s.d. of a partial solution to the final structure is calculated between C^α^ atoms in both models without applying superposition and excluding residues beyond 5.0 Å in distance. Models and maps were examined with *Coot* (Emsley *et al.*, 2010 ▸). Figures were prepared with *PyMOL* (Schrödinger).

### 2.3. Parameterization   

The *SHELXE* parameterization relies on the resolution-dependent defaults previously established for use in *ARCIMBOLDO* (Sammito *et al.*, 2016 ▸). For data sets with resolution better than 1 Å, 200 cycles of density modification with 0.5 density sharpening and 25% solvent content are set. For resolutions of 1.0–1.3 Å, 100 cycles of density modification with 0.25 density sharpening and 35% solvent content are set. For resolutions of 1.3–1.5 Å, 50 cycles of density modification with 0.1 density sharpening and 45% solvent content are set. For resolutions of 1.5–2.0 Å, 15 cycles of density modification with no density sharpening and 50% solvent content are set. For resolutions of 2.0–2.5 Å, ten cycles of density modification with no density sharpening and 60% solvent content are set. At worse than 2.5 Å resolution the previous parameterization is set, but this is still exploratory. By default, eight iterative autotracing cycles with a tenfold increase in the time dedicated to locating seeds including helical restraints are set. For the particular case of coiled coils (triggered when the coiled-coil mode is activated in *ARCIMBOLDO*), the helical restraints are changed to locate longer helices and the ‘free lunch’ is included with a resolution of 0.3 Å better than the actual data set, which was based on 150 test cases (Caballero *et al.*, 2018 ▸). The parameterization is complemented with information from the secondary-structure prediction or the homolog structure (adding -B1 for antiparallel β-sheets, -B2 for parallel or -B3 for both) and increasing the number of cycles until convergence.

Regarding refinement, three different, widely used programs are selected to illustrate the flexible integration of *SEQUENCE SLIDER*. Defaults for each of them are provided based on their documentation and defaults, which may be overridden by experienced users.

### 2.4. New structures and test data   

The characteristics of the data used in this study are summarized below and relevant statistics are given in Table 1 ▸. The data sets revisit the first novel structure solved with a prototype of the present implementation (MltC) and include a test case for each *SEQUENCE SLIDER* mode of use: lipase for the remote-homolog mode and FrmR E64H for the secondary-structure mode. A recent novel structure (PLP) determined using the latest implementation is also presented.

## 3. Algorithm description   

### 3.1. Proof of concept for *SEQUENCE SLIDER*: determination of the structure of MltC   

**Figure 1:**
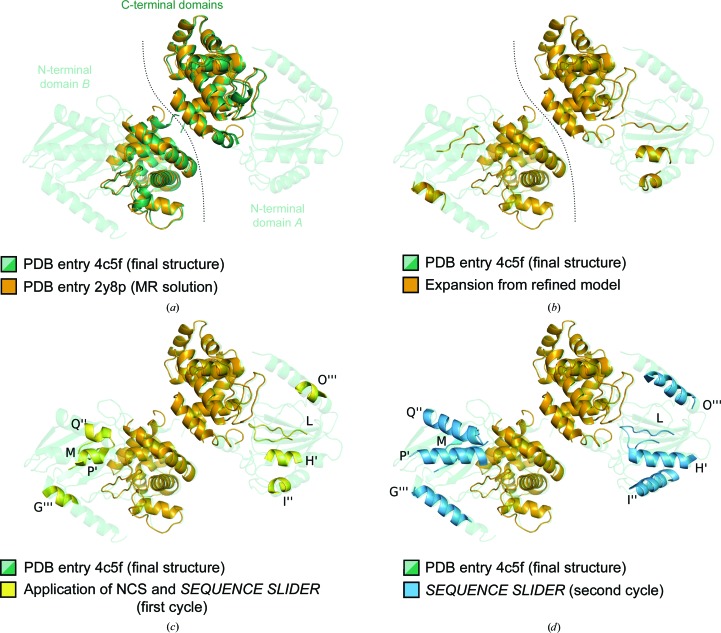
Structure solution of MltC (PDB entry 4c5f) using *SEQUENCE SLIDER*. (*a*) The final structure is shown in transparent green with a dashed line separating the two monomers. The molecular-replacement solution with the pruned MltE model (PDB entry 2y8p) corresponding to the C-terminal domain of MltC is shown in orange. (*b*) New fragments of the N-terminal domain obtained from the expansion of the refined solution after C-terminal side chains have been modelled and refined. (*c*) Further extension of the fragments in the N-terminal domain shown as yellow cartoons, derived from NCS. Related chains are marked with the same number of primes: *L*, *H*′, *I*′′ and *O*′′′ are equivalent to *M*, *P*′, *Q*′′ and *G*′′′, respectively. (*d*) The trace upon *SEQUENCE SLIDER* side-chain identification of sheets *L* and *M* allows the extension of the helices displayed in blue. From this point, *SEQUENCE SLIDER* can establish the sequence of helices *H*′ and *P*′, followed by chains *I*′′ and *Q*′′ and *G*′′′ and *O*′′′.

Proof of concept for *SEQUENCE SLIDER* came with the solution of the structure of MltC and the successful strategies were implemented in the distributed version of *SEQUENCE SLIDER* (Section 3.2). MltC is a soluble lytic transglycosylase from *Escherichia coli* and was solved with a data set that was isomorphous to the deposited 2.3 Å resolution data set (PDB entry 4c5f; Artola-Recolons *et al.*, 2014 ▸), which was not yet available at the time. Diffraction data to a lower resolution (2.7 Å) in the same *P*2_1_ space group with a dimer in the asymmetric unit were used. No model was known for the N-terminal domain of the protein, but a suitable model sharing 40% identity with the C-terminal domain of MltC was obtained by pruning the side chains and the first ten residues of the homologous protein MltE (PDB entry 2y8p; Artola-Recolons *et al.*, 2011 ▸). This template covers 54% of the MltC structure (green cartoon in Fig. 1 ▸ *a*) and turned out to have a similar structure within 0.8 Å r.m.s.d. calculated over 167 C^α^ atoms. A solution was found by *Phaser*, identified through a translation-function *Z*-score (TFZ) of 7.2 (orange cartoon in Fig. 1 ▸ *a*). The r.m.s.d. over 325 C^α^ atoms of the partial solution with two placed copies against the final structure was 1.6 Å. The electron-density maps derived from the partial solution failed to show any additional features outside the region of the fragment placed. Attempts to extend at a resolution of 2.7 Å with *SHELXE* were unsuccessful. In this case the parameterization used set ten cycles of density modification and ten iterative autotracing cycles with a 20-fold increase in the time dedicated to locate seeds, helical restraints at autotracing, a solvent fraction of 0.45, filling in missing data and extrapolating beyond the experimental resolution limit to 2.1 Å (*SHELXE* line -m10 -a10 -t20 -q -s0.45 -e2.1; Usón *et al.*, 2007 ▸). The (unpublished) prototype of *SEQUENCE SLIDER* run in 2011 used *BUSTER* version 1.6.0 (18 July 2009) and *SHELXE* version 2011/1.

The coordinates from the partial *Phaser* solution were characterized by a wMPE of 67.6° against the deposited structure, and 100 cycles of refinement with *BUSTER* (version 1.6.0; 18 July 2009) improved this by 3° (Table 2 ▸). This model did not provide sufficient starting information for *SHELXE* to reveal additional structural features, as observed by a decrease in the number of residues in the traced model (Table 2 ▸). Subsequently, modelling the side chains with *SCWRL*4 (Krivov *et al.*, 2009 ▸) according to the alignment from *HHpred* (Söding *et al.*, 2005 ▸) between the sequences of MltE and the C-terminal domain of MltC and refining them with *BUSTER* improved the phases to a wMPE of 47.0° (Table 2 ▸). Using the resulting map to provide initial phases, *SHELXE* traced several new fragments (Table 2 ▸ and Fig. 1 ▸ *c*): three independent helices containing nine, nine and eight residues with chain IDs *G*, *H* and *I*, respectively, and two similar strands containing eight and 12 residues named *L* and *M*, respectively.

**Figure 2:**
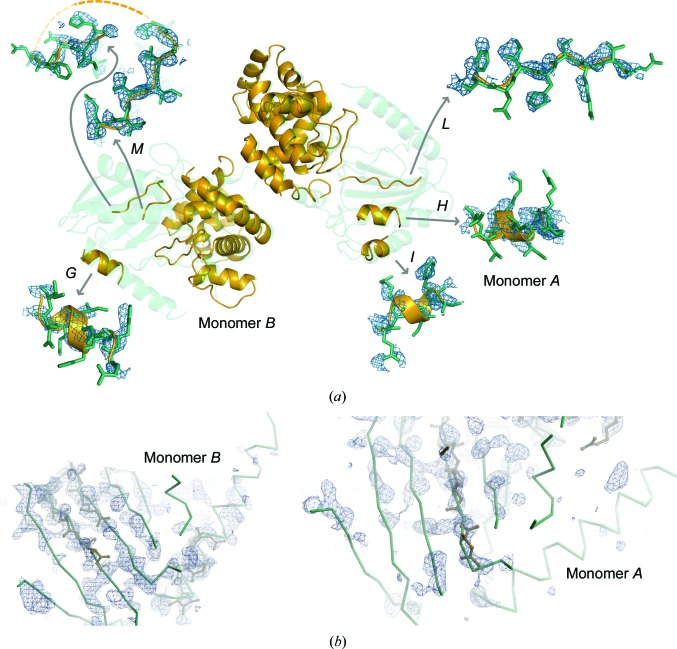
MltC structure and *F*_o_ maps contoured at 1.0σ generated from partial models after density modification at a resolution of 2.7 Å, revealing few additional features. (*a*) The final structure is shown as a green transparent cartoon and the partial structure is shown as an orange cartoon. The enlargement of the N-­terminal fragments shows the side chains in the final structure as green sticks. (*b*) N-terminal region with the partial solution shown as sticks and the final structure as a C^α^ trace with the electron-density map in blue for monomers *A* (right) and *B* (left).

Still, the electron-density map of the improved partial solution containing modelled side chains for the C-terminal domains and the new three helices and two strands did not show any additional features either for the side-chain atoms (Fig. 2 ▸ *a*) or the main-chain atoms (Fig. 2 ▸ *b*). Imposing the NCS derived from the two C-terminal domains deteriorated the tracing; nevertheless, it was possible to find the NCS relation between the N-terminal domains. Applying the operator relating the C-termini, four residues in each of the two strands (*L*+*M*) landed close to each other. A translation of 3.5 Å was required to superpose these four residues. The remaining new residues traced were also placed by this operation in positions supported by the electron density. They were further optimized by real-space rigid-body refinement. The map around the other monomer also corroborated the helices in the monomer thus assembled. Therefore, the five N-terminal fragments traced in the asymmetric unit were merged into four NCS-related chains per monomer: two β-strands connected by a small loop that summed 14 residues and three helices of seven, seven and six residues in length (yellow cartoons in Fig. 1 ▸ *c*).

In contrast to the C-terminal domain, for which a homolog was known and where its alignment to the target sequence provided the most probable sequence assignment, the fold of the N-terminal domain was unknown. Instead of trying out all amino-acid combinations allowed by the known sequence on the fragments of the trace, their secondary structure may be used as a restriction. Such an imposition is subject to the limitations in secondary-structure prediction from a sequence; its success in the absence of homologs is estimated to be limited to less than 80% (Jones, 1999 ▸).

**Figure 3:**
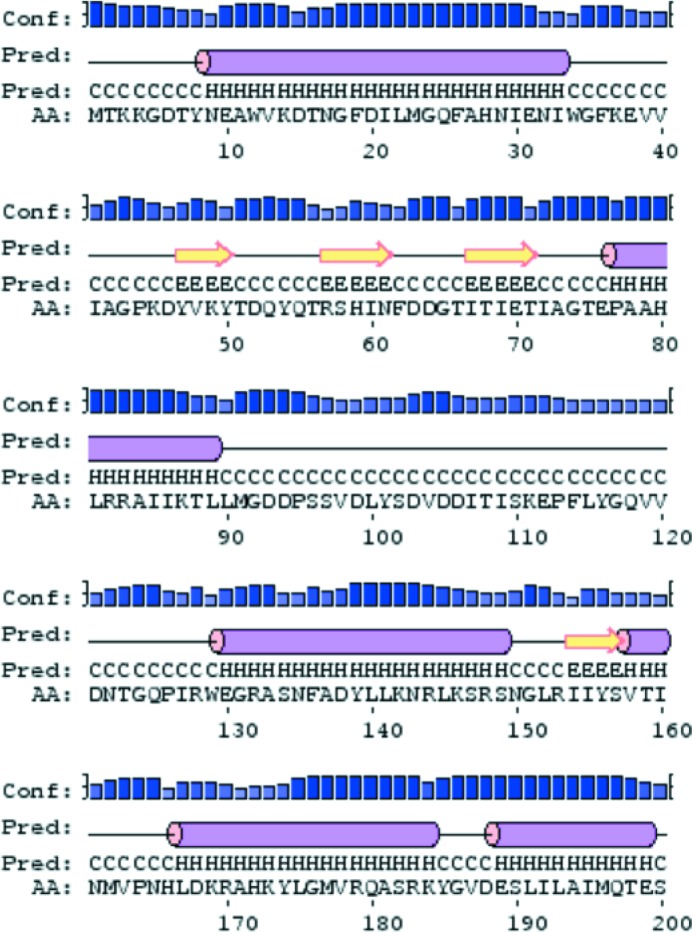
Secondary-structure prediction for the the MltC sequence using *PSIPRED* 3.0.

According to the secondary-structure prediction from *PSIPRED* 3.0 (Fig. 3 ▸), the missing N-terminal domain was expected to contain three strands with 4–5 residues each and three helices of 26, 13 and 20 residues. Sequence hypotheses were assigned to the N-terminal fragments traced, restricted by their secondary structure and tied to the NCS. As for the C-terminal domain, the different sequence hypotheses were assembled using *SCWRL*4 and the resulting models were refined using *BUSTER*. To allow some tolerance in secondary-structure tracing and prediction, sequence ranges were chosen that were three residues longer than those suggested by *PSIPRED* at both ends. For the strands, connecting loop residues were trusted and had their side chains modelled. For example, for the pairs of two strands traced, hypotheses could be restricted to span 14 residues comprising residues 47–61 or 57–71. Given a tolerance of three residues, they were set to cover residues 44–64 as well as 54–74.

Table 3 ▸ summarizes the indicators probed for the 15 alternative hypotheses generated for the strands. One of the assignments was discriminated from the rest based on the crystallographic indicators. Hypothesis 56–69 yielded *R* and *R*_free_ values that were over 1.4 percentage points better than the rest, as well as the top LLG (Table 3 ▸). The identity of *SEQUENCE SLIDER* hypotheses in the table was calculated as the number of residues in the partial trace assigned with the same amino acid as the corresponding residue in the final structure and with matching C^α^ atoms within 1.0 Å distance. Accordingly, a *SEQUENCE SLIDER* model with 100% identity would have all 682 residues correctly assigned and placed. At this stage, the *SEQUENCE SLIDER* input model started with an identity of 50.5% derived from the C-terminus. Hypothesis 56–69 raised it to 55.2%. This best-scored solution corresponded to the correct assignment.

The same procedure described for evaluation of the strands was applied in parallel to all other pairs of chains in the trace. According to the *PSIPRED* prediction (Fig. 3 ▸), the structure should contain the following helices: a 25-residue helix 1 (residues 9–33), a 13-residue helix 2 (residues 77–89) and a 20-residue helix 3 (residues 130–149). The smaller helix was compatible with helices *G*+*O*, *H*+*P* and *I*+*Q*, but the hypotheses rendered no discrimination in refinement indicators. The lack of discrimination was probably owing to the lower number of residues contained in the pair of helices at this stage (12 and 14 residues) along with their higher *B* factors, around 50 Å^2^, in comparison to the strands: 24 residues with an average *B* factor of 40 Å^2^ in the final structure.

Map improvement upon the addition of correct side-chain atoms on both strands allowed the extension in *SHELXE* of helices *G*+*O* and *H*+*P* to 15 residues each (shown as a sky blue cartoon in Fig. 1 ▸ *c*). In this extended trace, new sequence hypotheses were generated for the predicted helix 2 (residues 77–91 in Fig. 3 ▸) on the available NCS-related helix pairs *H*+*P*, *G*+*O* and *I*+*Q*. Hypothesis 77–91 modelled in *H*+*P* was distinguished by its figures of merit (FOMs; Table 4 ▸). It corresponded to the correct assignment and improved the global identity to 59.5%. Its sequence was fixed for the next round.

A fresh *SEQUENCE SLIDER* evaluation over the predicted helix 3 was performed on the remaining fragments (residues 167–184; Fig. 3 ▸) in chains *G*+*O* and* I*+*Q*. The latter was favoured by an improvement of 0.4% in *R*_free_ over the alternative hypotheses and similar or better FOMs (Table 5 ▸). It was confirmed as spanning residues 129–143, increasing the identity to 63.2%. From this point on, extension proceeded, rendering a fairly complete structure in which the sequence of the new residues was already known from the context of the initial, improved model.

#### 3.1.1. LLG as a figure of merit to score and filter hypotheses   

As seen from the values summarized in Tables 3 ▸, 4 ▸ and 5 ▸, the discrimination of the correct sequence assignments among alternative assigments is at best slim. Reviewing these results, none of the obvious refinement statistics, *R* factors and the real-space correlation coefficient (RSCC), allowed a clear distinction by itself. This is not unexpected given the small differences among models, the model bias given the still high overall errors and the possibility that some side chains are in incorrect conformations even if correctly assigned. Given sufficient computing resources, several possibilities can be committed to the next stage to be combined with all hypotheses for a new chain. Still, the problem remains as a complete combinatorial generation of solutions is not possible and a limit is necessary. We explored the possibility of reducing the number of solutions, filtering those with a more unfavourable free energy derived from the side chains, taking symmetry into account as calculated by *SCWRL*4. The data displayed in Tables 3 ▸, 4 ▸ and 5 ▸ shows that this would lead to discarding optimal solutions in the present case. The same result was found in other tests and the idea was abandoned.

**Figure 4:**
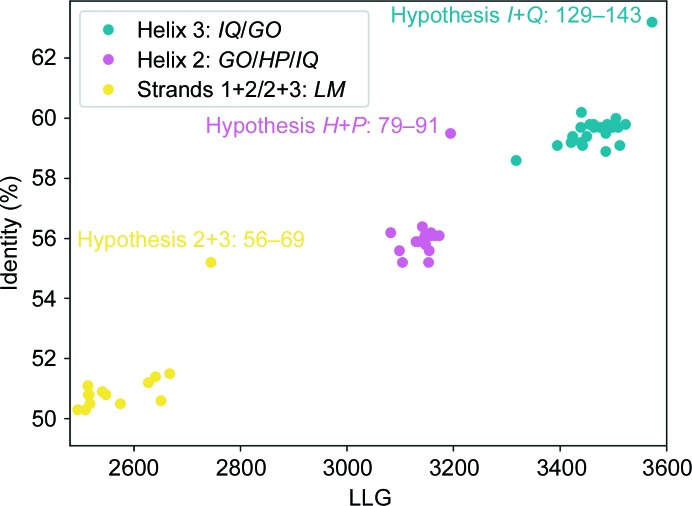
Identity of *SEQUENCE SLIDER* MltC models against their LLG score as sequence assignment progresses to a new fragment. The sequence hypotheses for the first, second and third *SEQUENCE SLIDER* cycles are shown as yellow, magenta and blue dots, respectively.

The *Phaser* LLG score appeared to be most sensitive for model evaluation. In our context, the LLG is not an absolute value as in *Phaser*, as it is calculated after coordinate and *B*-factor refinement of individual atoms. Still, all hypotheses are comparable as they share a common backbone and account for the same scattering fraction. For the *SEQUENCE SLIDER* run on the strands, the LLG scores for the different models ranged from 2460 to 2744 (Table 3 ▸ and Fig. 4 ▸). To evaluate the discrimination, we calculated the Δcontrast for a given LLG as the number of standard deviation from the mean LLG obtained for all models involving the same chain(s). For the strands, the top LLG-scored hypothesis, which is correct, has a contrast of 2.2σ and a difference from the second-best hypothesis of 1.0σ (Table 3 ▸). In the following evaluation for extended helices, the correct hypothesis for fragments *H*+*P* (residues 77–91) also scores the top LLG of 3194 (Table 4 ▸ and Fig. 4 ▸). Its contrast is 1.8σ and the difference from the second-best hypothesis is 0.8σ (Table 4 ▸). In the last evaluation step, the correct assignment for fragments *I*+*Q* (residues 129–143) again shows the top LLG of 3574 (Table 5 ▸ and Fig. 4 ▸). Its contrast is 2.2σ and the difference from the second-best hypothesis is 1.1σ (Table 5 ▸). In an iterative process, chains are assigned until no more discrimination is seen, opening the possibility of further improving the main-chain trace by expanding the best LLG-scored models with *SHELXE*.

#### 3.1.2. LLG as a figure of merit to prioritize fragments for *SEQUENCE SLIDER*   

Alternatively, to reduce calculations and avoid computing all possible sequences on each fragment, the polyalanine chains traced can be probed for their contribution to the LLG. This should score higher for larger fragments that are closer to the true structure and have lower *B* values in it (McCoy, 2017 ▸). Nevertheless, including the side chains in such fragments should have a higher effect on map improvement and therefore favour subsequent sequence identification and model extension.

The LLG contribution from each fragment pair to the whole trace was estimated as the difference between the LLG of the trace and the LLG of the trace omitting this fragment pair, calculated using a common r.m.s.d. value, VRMS refinement and *B* values set to 20 Å^2^ for all atoms in the chain to be probed.

The results in the case of the MltC solution are displayed in Table 6 ▸ and yield the same selection as found when probing all alternatives simultaneously. In the first *SEQUENCE SLIDER* run, fragments with strands (*L*+*M*) score highest, followed by helices *H*+*P*, *I*+*Q* and *G*+*O* (Table 6 ▸). In the original solution with the *SEQUENCE SLIDER* prototype, the strand fragments were first identified and assigned fixed sequences after side-chain probing, which allowed further extension of the helices. In the second *SEQUENCE SLIDER* cycle helices *H*+*P* score higher, followed by *G*+*O* and *I*+*Q*. In the third cycle after the *H*+*P* pair has had its sequence assigned, comparable values are obtained for *G*+*O* and *I*+*Q*. Therefore, the LLG contribution has been adopted to prioritize the order that *SEQUENCE SLIDER* will follow to probe side chains on fragments in the trace.

### 3.2. Algorithm   

**Figure 5:**
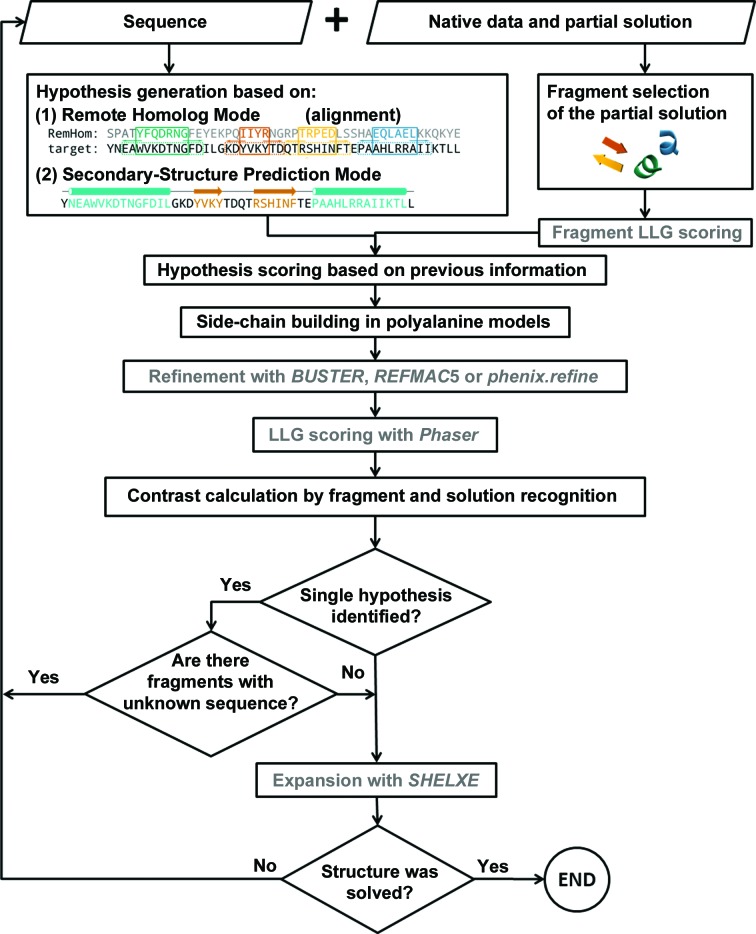
Program flow of *SEQUENCE SLIDER*. Input files provide, along with the diffraction data, the protein sequence, alignment and/or secondary-structure prediction and coordinates from partial solutions. The LLG contribution of each fragment guides selection for further steps. This information is combined to generate most probable hypotheses that are scored based on agreement with previous information. The sequence is modelled onto the trace. The resulting models are refined with either *BUSTER*, *REFMAC*5 or *phenix.refine* and are scored by the *Phaser* LLG. Once a single hypothesis is clearly favoured, it is fixed and the remaining fragments are re-evaluated in an iterative process until most of the possible sequence match is retrieved. The best-scored models are then submitted to expansion with *SHELXE*. Steps from external programs are coloured in grey.

*SEQUENCE SLIDER* aims to improve a stalled *ARCIMBOLDO* solution by trialling different side-chain assignments and sending multiple models to refinement. The best LLG-scored model(s) are sent to a fresh expansion attempt through density modification and tracing. The program flow is summarized in Fig. 5 ▸ and each step is described below.

#### 3.2.1. Input   

*SEQUENCE SLIDER* requires a native diffraction data set (in HKL and MTZ format), a sequence (FASTA format), a partial *ARCIMBOLDO* or MR solution (PDB format) and an instruction file with extension .bor, which contains the parameterization of the run (‘Sequence’ and ‘Native data and partial solution’ boxes in Fig. 5 ▸). If the partial solution has been generated using a remote homolog, its alignment to the target sequence should be provided (PIR format). Otherwise, secondary-structure prediction should be supplied (*PSIPRED* format). Most parameters have suitable defaults. The only mandatory input is the data description, which includes MTZ labels, the number of molecules per asymmetric unit and the molecular weight. NCS may be specified. As a prior step, the dependencies and input files are verified.

#### 3.2.2. Generation and selection of hypotheses   

*SEQUENCE SLIDER* provides alternative paths depending on the available hardware. In its lightest version, suitable for a single machine, serine is modelled at every residue rather than generating multiple hypotheses based on the sequence. This general extension may suffice in easier cases and has been discussed in previous literature (Schwarzenbacher *et al.*, 2004 ▸). For challenging cases that are stuck in tracing, the *SEQUENCE SLIDER* grid version, as described for the solution of MltC, generates hypotheses matching information from the sequence and structure. In the most computationally expensive method all residues are modelled; alternatively, assignment may be limited to hydrophobic side chains, saving 20% of the execution time owing to the reduction in the number of parameters being refined. As seen, for instance, in the case of MltC (Fig. 2 ▸), such residues tend to show higher side-chain density than the generally more exposed polar residues. Other methods, such as *Sculptor* (Bunkóczi & Read, 2011 ▸), *CHAINSAW* (Stein, 2008 ▸) and model preparation in *MOLREP* (Lebedev *et al.*, 2008 ▸), also offer a range of choices to truncate side-chain atoms in a model.

*SEQUENCE SLIDER* classifies continuous fragments on the partial model to which a sequence will be assigned. If more than one polypeptide chain is present in the model, variations in their sequence may be probed simultaneously, as long as their combination does not exceed a hard limit (1000). Otherwise, hypotheses are tested separately, fragment by fragment, following their calculated contribution to the total LLG (Section 3.1.2; Fig. 5 ▸). In the presence of NCS, identical chains should be supplied by the user and matching hypotheses will be generated simultaneously. The program will check that their geometry and size are equivalent.

Hypothesis generation integrates the available previous knowledge on the target model. If a partial solution comes from a remote homologous structure or a partial trace can be matched to the fold of a homolog, the correspondence from their sequence alignment will be used to restrict the models to be tested [‘Hypothesis generation based on: (1) Remote Homolog Mode’ in Fig. 5 ▸]. In the more general case only the secondary-structure prediction is used [‘Hypothesis generation based on: (2) Secondary-Structure Prediction Mode’ in Fig. 5 ▸].

#### 3.2.3. Generation and selection of hypotheses: Remote Homolog Mode   

Using the remote homolog, the fragments on the protein model are classified based on contiguous residues, ignoring their secondary structure. Additional sequence hypotheses are generated by sliding the alignment provided. The scoring function favours hypotheses in which the side-chain assignment agrees with previous information and penalizes deviations (‘Hypotheses scoring’ in Fig. 5 ▸). In Remote Homolog Mode, the score of a hypothesis is 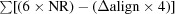, where NR is the number of residues in a chain and Δalign is the number of residues by which a fragment is shifted from the original alignment. The sum is over all chains being modelled. Therefore, deviation from the original alignment is penalized and extensive assignments are prioritized. In the context of *Sculptor* (Bunkóczi & Read, 2011 ▸), *CHAINSAW* (Stein, 2008 ▸) or model preparation in *MOLREP* (Lebedev *et al.*, 2008 ▸), it is logical to use a single possibility given by the alignment to prepare an MR search template. In our context, template-model decomposition and rigid-group refinement against the rotation function (*gyre*) and after translation (*gimble*) (McCoy *et al.*, 2018 ▸) may shift secondary-structure elements as rigid groups (Millán *et al.*, 2018 ▸). The structural displacements introduced may break the original correspondence of fragments, which should be recovered by sliding the alignment.

#### 3.2.4. Generation and selection of hypotheses: Secondary-Structure Prediction Mode   

In the absence of prior information regarding the sequence of a fragment, hypothesis generation is restricted by matching its secondary structure to that expected from the sequence as predicted using *PSIPRED* (Jones, 1999 ▸). *SEQUENCE SLIDER* organizes this information in contiguous residues of common secondary structure. The secondary structure of each residue in the trace is assigned using *ALEPH* (Medina *et al.*, 2020 ▸; ‘Fragment extraction of the partial solution’ in Fig. 5 ▸). *ALEPH* classifies residues into α-helices and β-strands or coil based on the backbone geometry and the environment of overlapping tripeptides as described by characteristic vectors (Sammito *et al.*, 2016 ▸). Loop residues may be retained or excluded from modelling. *SEQUENCE SLIDER* then generates hypotheses, restricted by the secondary-structure match, sliding the sequence over the fragments in the trace and scoring them by the agreement between the predicted secondary structure and that found in the fragment. A hypothesis is scored as the sum of scores for all individual residues assigned: for modelled residues this is 6 if the secondary-structure prediction matches the fragment and −4 otherwise. Residues at the edges of the polypeptide chain are not modelled and their scores are reduced to 4 and −1, respectively. To account for uncertainties in secondary-structure assignment on sequence and traces, a tolerance may be set to extend sliding over the edges by the specified number of residues but introducing a penalty in the score to keep track of this deviation. By default, *PSIPRED* confidence levels are ignored.

#### 3.2.5. Modelling hypotheses   

After sequence hypotheses have been generated, side chains are incorporated into the model. The tests described use *SCWRL*4 (‘Side-chain building in polyalanine models’; Fig. 5 ▸), which uses a backbone-dependent rotamer library, soft van der Waals atom–atom interaction potentials, fast collision detection and crystal symmetry (Krivov *et al.*, 2009 ▸) to obtain the most probable conformation. In its distributed implementation, side chains are built, without further dependencies, using one of *ARCIMBOLDO*’s ancillary programs (*SPROUT*).

Models with random sequence are included in the pool and are scored to provide a baseline (Caballero *et al.*, 2018 ▸). The new models assembled are sent to refinement (‘Refinement with *BUSTER* or *REFMAC*5 or *phenix.refine*’ in Fig. 5 ▸), distributed on a grid if available. Ideally refinement should be parameterized by the user, but our general defaults for the refinement of partial models are provided. With *BUSTER*, models are submitted to ten (big) cycles of refinement with no water addition and using rigid bodies, one single thread and NCS if present (-noWAT -nbig 10 -RB -nthread 1 -autoncs). With *REFMAC*5, models are submitted to 100 cycles of refinement with jelly-body restraints (0.02). With *phenix.refine*, models are submitted to ten cycles of refinement with secondary-structure and Ramachandran restraints.

Models are scored based on their calculated LLG (‘LLG scoring with *Phaser*’ in Fig. 5 ▸). The contrast for a given LLG is calculated as the number of standard deviation from the mean LLG obtained for all models involving equivalent hypotheses to find the most probable or to distinguish the correct hypothesis (‘Contrast calculation by fragment and solution recognition’ in Fig. 5 ▸). *SEQUENCE SLIDER* fixes this assumption for the following steps (diamond box in Fig. 5 ▸). If the evaluation was inconclusive, side-chain assignment on this fragment is delayed and evaluation proceeds to the next round with another available fragment (left arrow leaving the diamond box in Fig. 5 ▸). After all fragments have been probed, *SEQUENCE SLIDER* selects the best models and/or their corresponding maps based on the highest LLG and submits them to *SHELXE* expansion (‘Expansion with *SHELXE*’ in Fig. 5 ▸). The *SHELXE* parameterization relies on the same resolution-dependent defaults previously established for use in *ARCIMBOLDO* (Section 2.3). If the phases improve, the rest of the structure is revealed by *SHELXE* and identified by the number of residues and CC in the final trace. While no clear solution is obtained, *SEQUENCE SLIDER* continues to iterate, generating hypotheses for the new fragments traced after they are merged into the initial model. As sequence is assigned to fragments, their NCS relationships are derived from the sequence and matching fragments are extended. In subsequent *SEQUENCE SLIDER* cycles, NCS-related fragments are evaluated simultaneously and restricted to have the same sequence assignment.

## 4. Discussion and examples   

### 4.1. Lipase/acylhydrolase structure   

**Figure 6:**
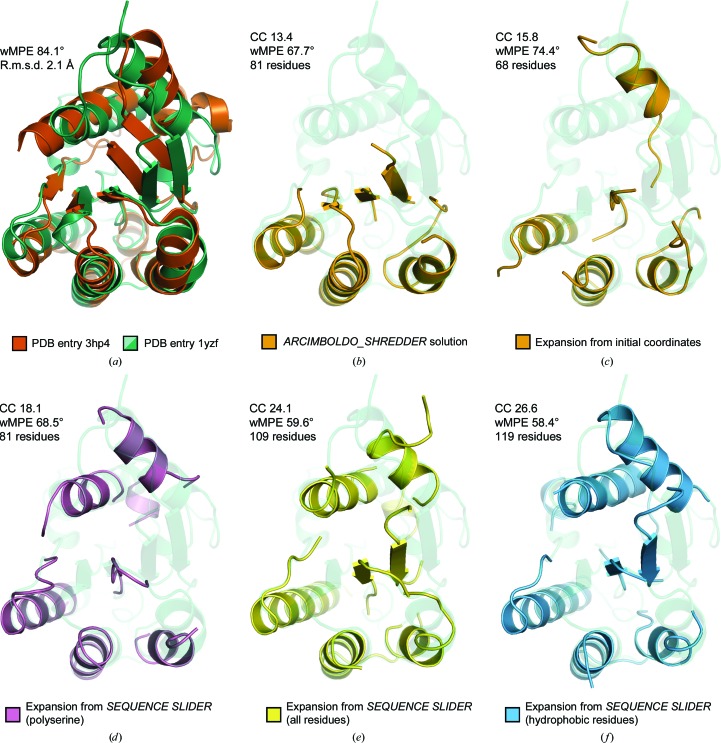
Solution stages and phasing tests for lipase using the PDB entry 3hp4 template. (*a*) Superposition of target (green cartoon; PDB entry 1yzf) and search-template (vermilion) structures. (*b*) The *ARCIMBOLDO_SHREDDER* partial solution is shown as an orange cartoon. (*c*) The trace resulting from the partial solution in (*b*) deteriorates with respect to the initial model. (*d*) Tracing from a refined polyserine model. (*e*) The best *SEQUENCE SLIDER* trace after modelling all residues. (*f*) The best *SEQUENCE SLIDER* trace after modelling only hydrophobic residues.

To exemplify the Remote Homolog Mode using *phenix.refine* as a refinement program, we applied *SEQUENCE SLIDER* to a partial solution obtained by *ARCIMBOLDO_SHREDDER* (Millán *et al.*, 2018 ▸). The target is lipase/acylhydrolase from *Enterococcus faecalis*, which displays a Rossmann fold with 195 residues, 36% solvent content and 1.9 Å resolution (PDB entry 1yzf; Midwest Center for Structural Genomics, unpublished work; Table 1 ▸). *ARCIMBOLDO_SHREDDER* was able to find a partial solution for this structure using a homolog from *Pseudoalteromonas* sp. (PDB entry 3ph4; Jung *et al.*, 2011 ▸) that shares 22% identity and shows an r.m.s.d. of 2.3 Å over 161 C^α^ atoms of matching secondary structure (Fig. 6 ▸ *a*). As a matter of comparison, the phases derived from PDB entry 3ph4 superposed on PDB entry 1yzf are characterized by a wMPE of 75.9°. Unsurprisingly, no correct solution was found using this whole template as search model for MR. The *ARCIMBOLDO_SHREDDER* run with default settings found almost 630 rotations that were grouped into 15 clusters. A correct solution, composed of 81 residues divided into three helices and four strands (Fig. 6 ▸ *b*), was distinguished from the rest after rigid-body refinement. Its LLG, TFZ and initial CC were 56, 10.4 and 13.4%, respectively, while the LLG, TFZ and initial CC values within other clusters did not reach values above 44, 8.0 and 11.2%, respectively. *SHELXE* ran 15 cycles of density modification and 25 autotracing iterative cycles with secondary-structure and tertiary-structure restrained autotracing of helices and parallel β-strands with a solvent fraction of 0.5. The reflection file contained amplitudes (*SHELXE* line -m15 -a25 -q -B2 -s0.5 -v0 -f). Autotracing was only able to reveal an additional helix and lost the β-strands in the original fragment, scoring a maximum CC of 15.8, calculated from 68 residues, and wMPE of 74.4°. Autotracing of wrong solutions reached CC values of around 11%. *SEQUENCE SLIDER* rescued this solution, where expansion was otherwise unsuccessful.

The best-scoring partial solution from *ARCIMBOLDO_SHREDDER* was composed of five polyalanine fragments: (i) a coil and a three-residue strand (residues 2–9 in PDB entry 3ph4), (ii) a coil and a ten-residue helix (residues 52–70), (iii) a seven-residue strand (residues 76–82), (iv) a 15-residue helix connected by a coil to a five-residue strand (residues 92–119) and (v) a 14-residue helix and coil (residues 130–148). Of these 81 residues, 23 were not within 1 Å of the final structure. We applied *SEQUENCE SLIDER* using *phenix.refine* with the alignment between template and target sequence from *HHpred* (Söding *et al.*, 2005 ▸). We set the sliding tolerance to 1, generating three hypotheses for each one of the five fragments present in the partial solution, totalling 243 sequence possibilities. As a baseline, we included ten random sequence hypotheses.

**Figure 7:**
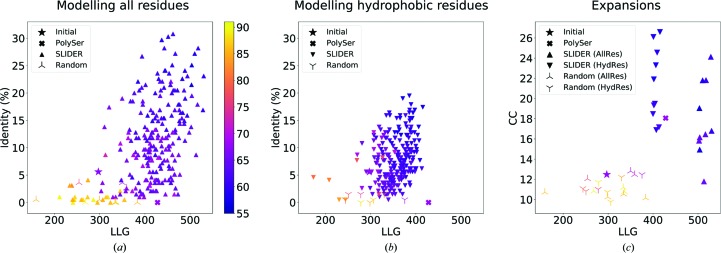
Identity, wMPE and CC versus LLG scoring of *SEQUENCE SLIDER* (SLIDER) models generated from a partial solution for lipase. (*a*) Hypotheses from modelling all residues (AllRes) are shown as triangles and initial and polyserine (PolySer) models are shown as stars and crosses, respectively; random models are shown as propellers. (*b*) Modelling only hydrophobic residues (HydRes) shown as triangles. (*c*) Expansion results for the top ten LLG models from the *SEQUENCE SLIDER* runs in (*a*) and (*b*).

The original *ARCIMBOLDO_SHREDDER* structure with no coordinate modifications (PolyAla) rendered a refined structure characterized by an LLG of 297 and a wMPE of 71.4° (stars in Fig. 7 ▸ *a*), whereas the wMPE from the electron-density map (wMPE*) was 67.5°. In the lighter *SEQUENCE SLIDER* version, which models a serine in each residue, the PolySer refined structure possessed a better LLG of 428 but had a similar wMPE to the previous model (crosses in Fig. 7 ▸ *a*). In the default version, modelling all residues, the sequence hypotheses ranged in identity from 0 to 30% and the LLG ranged from 210 to 530 (triangles in Fig. 7 ▸ *a*). The ten best-scored LLGs corresponded to models having the lowest initial wMPE against the final structure, from 62.7 to 68.9°. Maps from such models rendered a somewhat lower wMPE*, ranging from 56.6 to 64.8°. The models generated incorporating only hydrophobic residues gave poorer values in comparison to the full version as fewer residues were being assigned, reaching a maximum identity and LLG of 19% and 415, respectively (triangles in Fig. 7 ▸ *b*). On the other hand, the ten best-scored LLGs in this last run already provided a significant phase improvement, with a wMPE of 64.3–67.6° and a wMPE* of 57.7–61.4°. The statistics for the models from random sequences were worse than the initial input, with values that did not reach 3% identity, an LLG of 384, a wMPE of 72.7° and a wMPE* of 69.1°.

The resulting trace of the refined PolyAla model deteriorated upon refinement, as seen by the increase in its wMPE to 74.4° (Fig. 6 ▸ *c*), which was also the case for models generated from random sequences (propellers in Fig. 7 ▸ *c*). In contrast, the PolySer trace revealed two correct 11-residue helices and was characterized by a CC of 18.1 and a wMPE of 68.5° (Fig. 6 ▸ *d*). Upon the modelling of all side chains, the improvement in the trace revealed the rest of the structure (triangles in Fig. 7 ▸ *c*). In the run modelling all residues, the best trace was characterized by a CC of 24.1% with 109 residues and a wMPE of 59.6° (Fig. 6 ▸ *e*). Finally, the run modelling only hydrophobic residues rendered a best trace characterized by a CC of 26.6%, 119 residues traced and a wMPE of 58.4° (Fig. 6 ▸ *f*).

The results on this borderline case suggest that in easier cases extending the polyalanine trace to polyserine may suffice and that the incorporation of hydrophobic residues may introduce fewer errors as they typically adopt fewer conformers and are more restricted by neighbouring fragments.

### 4.2. Coiled-coil structure: FrmR E64H   

**Figure 8:**
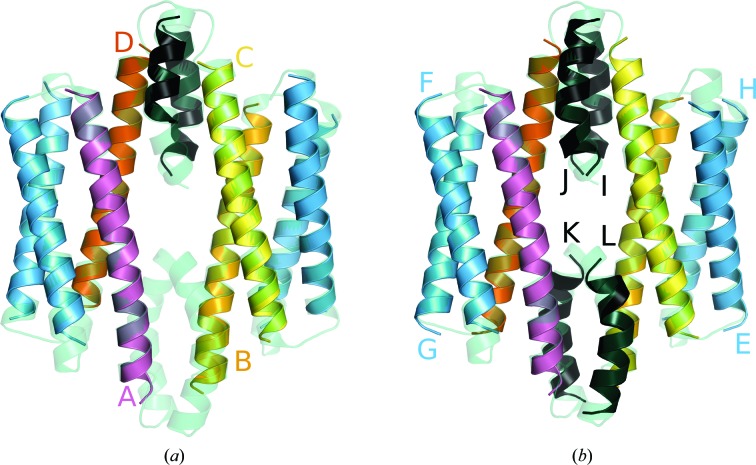
Phasing and sequence assignment for PDB entry 5lcy. The final structure in shown as a green transparent cartoon and the traced chains *A*, *B*, *C*, *D*, *E*+*F*+*G*+*H* and *I*+*J* from the partial solution are coloured purple, orange, yellow, red, blue and black, respectively. (*a*) *ARCIMBOLDO_LITE* solution. *SEQUENCE SLIDER* assembles the correct sequence independently for chains *A*, *B*, *C* and *D*, which allows the discovery of NCS-related copies that lead to structure completion. (*b*) Finally, the sequence for the last helix (69–83), coloured black, is assigned.

Phasing coiled coils is frequently a challenge. Their diffraction data are usually anisotropic and dominated by modulation, as even in the absence of true translational NCS (Read *et al.*, 2013 ▸) the periodic helices typically adopt preferred directions in the crystal. FrmR E64H is a coiled coil with 91 residues and was solved using *ARCIMBOLDO_LITE* (PDB entry 5lcy; Osman *et al.*, 2016 ▸), containing four monomers in the asymmetric unit and a solvent content of 42% (Table 1 ▸). Here, we replicated its solution using three polyalanine helices of 18 residues with the recently optimized coiled-coil mode in *ARCIMBOLDO* (Caballero *et al.*, 2018 ▸), which incorporates suitable defaults, inversion of helix direction and a verification step, as for coiled coils discriminating a true solution among pseudo-solutions may be challenging. A single rotation cluster was found in the first fragment search with a top LLG of 74. Two rotation clusters were found in the second fragment search with top LLGs of 203 and 158. In the third fragment search, three clusters sharing similar LLGs of around 240 were found to contain partial correct solutions. Default *SHELXE* parameterization from *ARCIMBOLDO* coiled-coil mode was used (Caballero *et al.*, 2018 ▸), ten cycles of density modification and eight iterative autotracing cycles with a tenfold increase in the time dedicated to locate seeds, long helical restraints at autotracing, a solvent fraction of 0.6, filling in missing data and extrapolating beyond the experimental resolution limit to 1.82 Å (*SHELXE* line -m10 -a8 -t10 -Q -s0.6 -e1.82; Usón *et al.*, 2007 ▸). Finally, four partial solutions led upon expansion to a CC of above 40%. As for other structures with strong modulation of the data, in the case of coiled coils or nucleic acids, wrong solutions may be characterized by extremely high CC values, even above 40%, and successful side-chain discrimination may be used to disambiguate. In this case the top solution had a CC of 45.3% and was composed of nine unconnected helices that summed 240 residues, corresponding to 64% of the complete main chain in the structure (Fig. 8 ▸ *a*).

*PSIPRED* predicts three helices composed of 26, 31 and 19 residues, separated by 4–6 coil residues. We used *SEQUENCE SLIDER* to assemble hypotheses restricted by the secondary-structure prediction on the helices given a tolerance of two residues (Table 7 ▸). The complete initial model containing 240 residues divided into ten chains possessed an LLG of 1710 given a VRMS of 0.17, which increased to 2713 upon structure refinement. The LLG contributions for the ten chains varied from 92 to 400. We applied *SEQUENCE SLIDER* to the chains with an LLG contribution of above 350 (chains *A*, *B*, *C* and *D*; Table 7 ▸).

**Figure 9:**
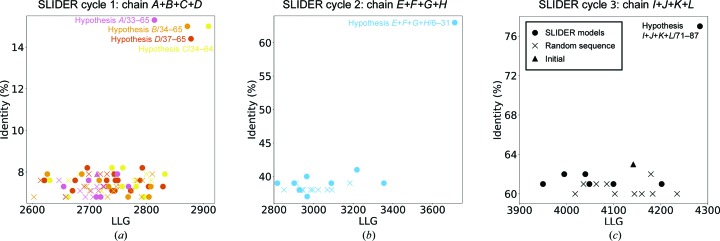
Graphs showing identity versus the LLG score for the different hypotheses generated by *SEQUENCE SLIDER* (SLIDER) for PDB entry 5lcy. The scale difference is owing to each cycle building upon the previous model. (*a*) First *SEQUENCE SLIDER* cycle, whereby the correct sequence assignment is distinguished for chains *A*, *B*, *C* and *D*, shown in magenta, orange, yellow and red, respectively. (*b*) The correct sequence is assigned for the NCS-related chains *E*+*F*+*G*+*H*. (*c*) The correct sequence is assigned for the NCS-related chains *E*+*F*+*G*+*H*. *SEQUENCE SLIDER* models and models with random sequence are represented by circles and crosses, respectively.

Chain *A*, with an LLG of 406, was a 33-residue helix, which should be matched to the longest predicted helix of 31 residues. The LLG for the seven models generated ranged from 2820 to 2645 (Fig. 9 ▸ *a*). The top hypothesis 33–65 was distinguished from the others by a difference in contrast of 1.2σ. The second-best LLG of 374 corresponded to chain *B*, another long helix composed of 32 residues, which could only be matched to the second helix in the sequence. The eight hypotheses generated rendered LLGs from 2870 to 2600. The top hypothesis 34–65 was prominent, with a difference in contrast of 1.0σ. Chain *C* gave an LLG of 355, and being a 31-residue helix it must also correspond to the long second helix. The nine hypotheses derived gave LLGs from 2910 to 2630. Hypothesis 34–64 was distinguished from the others by a difference in contrast of 1.2σ. Chain *D*, the LLG contribution of which was 356, was composed of a 29-residue helix and thus was compatible with either the first or the second helix. This led to six hypotheses from the 26-residue predicted first helix and 11 hypotheses from the 31-residue predicted second helix, with LLGs ranging from 2880 to 2615. Hypothesis 37–65 was distinguished from the others by a 0.9σ difference in contrast.

From the results summarized in Table 7 ▸, we identified the correct hypotheses based on their contrast for chains *A*, *B*, *C* and *D*. Fixing these four chains establishes their NCS relation, which was used in the following rounds. Furthermore, we merged fragments to have, per monomer, a 33-residue helix, a 26-residue helix and a final 12-residue helix, which was missing for two monomers (Fig. 8 ▸ *b*).

NCS-matching assignments on the 26-residue helices present in chains *E*+*F*+*G*+*H* generated nine hypotheses from the sequence-predicted helix 1. These models yielded LLGs of 2745–3710 (Fig. 9 ▸ *b*). Hypothesis 6–31 was identified as correct through its Δcontrast of 2.2σ (Table 7 ▸). The remaining predicted helix 3 and chains *I*+*J*+*K*+*L* generated 11 hypotheses. We identified hypothesis 11–27 as the correct hypothesis with a more modest contrast difference of 0.6σ but scoring top on all other FOMs as well (Fig. 9 ▸ *c*). In all cases, the LLG reached by models of random sequence served to establish the LLG range corresponding to models with the incorrect sequence assignment.

### 4.3. Novel lipoprotein structure   

**Figure 10:**
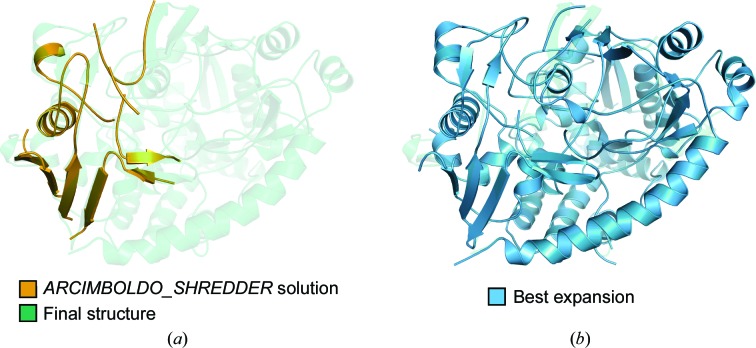
Phasing PLP. (*a*) The *ARCIMBOLDO_SHREDDER* solution is shown as an orange cartoon with the final structure in transparent green. (*b*) The best *SEQUENCE SLIDER* trace is shown in blue.

PLP is a novel lipoprotein of 659 residues from the human pathogen *Streptococcus pneumoniae*, which was phased using *SEQUENCE SLIDER*. It is an extracellular solute-binding protein that belongs to the SBP-bac_5 family (Pfam PF00496). Its mature form is 638 residues in length after the proteolytic cleavage of its N-terminal signal peptide. The protein is composed of three domains with a mixed contribution of α-helices and β-strands. The data set reaches high resolution (1.26 Å) but with low completeness (37%), and for shells up to 2.0 Å resolution the completeness is limited to 90% (Table 1 ▸). The structure of a transport protein from *Bacillus anthracis* (PDB entry 6npo; Center for Structural Genomics of Infectious Diseases, unpublished work), with an identity of 26%, was identified using *HHpred* (Söding *et al.*, 2005 ▸). This model rendered a solution using *ARCIMBOLDO_SHREDDER* (Fig. 10 ▸ *a*), set to preserve coil regions and extract 522 spherical models with an eLLG of 30, corresponding to a size range of 97–101 amino acids. One of the four rotation clusters stood out for its higher FOMs, with a top TFZ of 7.3 against 6.12 and a top LLG of 55.3 against 36.7 for the second-best cluster. *SHELXE* ran ten cycles of density modification and 18 autotracing iterative cycles with secondary-structure and tertiary-structure restrained autotracing of helices and β-strands and a solvent fraction of 0.45 (*SHELXE* line -m10 -a18 -q -s0.45 -B3; Usón *et al.*, 2007 ▸). *SHELXE* tracing was unsuccessful, even with combination of phases with *ALIXE* (Millán *et al.*, 2020 ▸), and in this case *SEQUENCE SLIDER* was essential in solving the structure of PLP.

**Figure 11:**
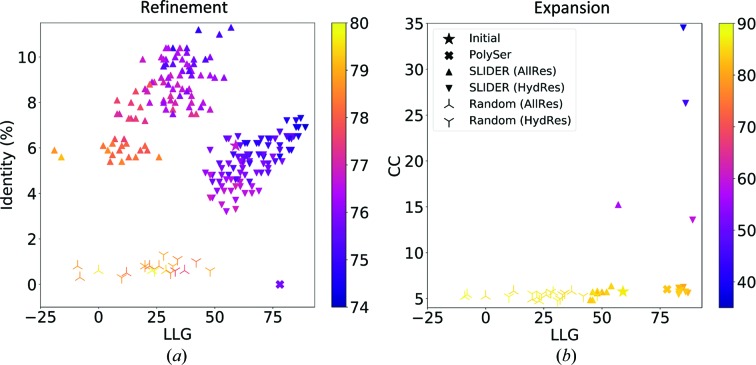
Characterization of PLP *SEQUENCE SLIDER* models. (*a*) Refinement statistics modelling all residues (triangles pointing upwards) and modelling only hydrophobic residues (triangles pointing downwards). (*b*) Expansion statistics for the ten top LLG models in each run. Models are coloured according to wMPE.

We used *BUSTER* (Bricogne *et al.*, 2018 ▸) to refine side-chain extended models, but the default ten cycles did not reach convergence and the positions of some fragments changed drastically. Therefore, we evaluated the models rendered by different numbers of refinement cycles with the *Phaser* LLG and the CC in *SHELXE* (Table 8 ▸). The LLG and CC indicated two cycles as the optimum. Accordingly, we changed the *SEQUENCE SLIDER* default parameterization and reran it modelling all residues and reducing complexity to model only hydrophobic residues (Fig. 11 ▸). Modelling only hydrophobic residues provided higher LLGs and their resulting expansion (the ten best LLG models) was also better. Given sufficient cycles, the correct model with all side chains would also have converged into a solution. Its expansion from the run modelling all residues was set apart from the rest with a CC of 15.3 compared with the others, which were below 7 (Fig. 11 ▸ *b*). On the other hand, the best expansion from the hydrophobic residues run already managed to reveal almost the entire structure (Figs. 10 ▸ *b* and 11 ▸ *b*); it had a CC of 34.5 with 600 traced residues (96% of the whole structure). Models from random sequences behaved like mismatched models.

## 5. Concluding remarks   

Extending partial polyalanine solutions with side chains modelled covering a range of possible assignments may allow the solution of partial solutions from *ARCIMBOLDO* that would otherwise fail. The procedure implemented in *SEQUENCE SLIDER* involves deriving possible hypotheses compatible with prior information, generating the extended models and refining them. The previous knowledge used is the alignment to a homolog if available and/or the secondary-structure prediction. LLG scoring is used both to guide the choice of fragment to be extended and to select the refined models to be combined in a fresh round of fragment extension. Models with random sequence assignment are generated and included in the pool to provide a baseline. In no case did such models lead to a solution. When no clear path for model completion is apparent, the models are subject to expansion through density modification and autotracing, and solutions can be recognized by the CC of the final traces. In simpler cases, a light version extending every fragment as polyserine may suffice, whereas in challenging cases a finer side-chain assignment is required. This can be extended to all side chains or limited to hydrophobic residues, which tend to have lower *B* factors and favour fewer rotamers than polar side chains. The *SEQUENCE SLIDER* method, which is available through the *ARCIMBOLDO* distribution, has been instrumental in solving new protein structures.

## Figures and Tables

**Table 1:** Summary of data sets ASU, asymmetric unit.

Protein	MltC	Lipase	FrmR E64H	PLP
PDB code	4c5f	1yzf	5lcy	
Resolution (Å)	2.7	1.9	2.1	1.3
Space group	*P*2_1_	*P*3_2_21	*P*2_1_	*P*2_1_2_1_2_1_
Unit-cell parameters				
*a* (Å)	49.78	45.92	68.96	102.70
*b* (Å)	113.03	45.92	25.70	107.57
*c* (Å)	60.99	148.03	100.79	56.60
β (°)	92.88		103	
Residues in ASU	684	195	364	638
Monomers in ASU	2	1	4	1
Solvent content (%)	47	36	42	35
Partial solution	MR	*ARCIMBOLDO_SHREDDER*	*ARCIMBOLDO_LITE*	*ARCIMBOLDO_SHREDDER*

**Table 2:** Summary of extension steps and tests applied to the partial solution of MltC The abbreviations used are wMPE, weighted mean phase error between the phases calculated from the model and from the deposited structure; wMPE*, wMPE where phases are calculated using the map instead of the coordinates of a partial model; #Res/atoms, number of residues/atoms present in the model; LLG, log-likelihood gain; CC, correlation coefficient.

	Initial model		Refined model		Trace using map from refinement			
Strategy	wMPE (°)	#Res/atoms	LLG	wMPE* (°)	CC	#Res/atoms	wMPE (°)	LLG
*Phaser* solution	67.6	354/1764	852	64.5	23.4	322/1520	65.2	379
*SEQUENCE SLIDER*	64.3	354/2764	1974	47.0	30.0	387/1870	51.8	598

**Table 3:** Refinement statistics of *SEQUENCE SLIDER* hypotheses generated for β-strands in chains *L* and *M* of the partial model of MltC (sequences 44–65 and 55–73) HypSeq, sequence hypothesis; RSCC, real-space correlation coefficient; mc, main-chain atoms; sc, side-chain atoms; LLG, log-likelihood gain; wMPE, weighted mean phase error; Energy, side-chain free energy as calculated with *SCWLR*4 as described in Krivov *et al.* (2009 ▸). Δcontrast is the number of standard deviations that an LLG is above the average LLG for all models involving equivalent hypotheses. wMPE* is calculated from the phases from the map from refinement rather than from coordinates.

HypSeq	*R*	*R*_free_	RSCCmc	RSCCsc	Energy	LLG	Δcontrast	wMPE (°)	wMPE* (°)	Identity (%)
56–69	0.3671	0.4060	0.893	0.768	1097	2744	2.2	43.6	43.1	55.2
47–60	0.3813	0.4215	0.892	0.762	1119	2667	1.2	44.5	43.9	51.5
55–68	0.3921	0.4293	0.890	0.765	986	2650	1.0	44.8	44.1	50.6
57–70	0.3943	0.4256	0.885	0.758	987	2640	0.9	44.9	44.4	51.4
44–57	0.3999	0.4395	0.893	0.766	1079	2627	0.7	45.0	44.5	51.2
45–58	0.4129	0.4470	0.885	0.756	1015	2575	0.1	45.8	45.2	50.5
48–61	0.3964	0.4288	0.876	0.753	997	2547	−0.3	45.2	44.6	50.8
46–59	0.4277	0.4596	0.871	0.752	1033	2540	−0.3	45.5	44.9	50.9
60–73	0.4124	0.4453	0.881	0.758	960	2517	−0.6	45.1	44.6	50.5
49–62	0.4121	0.4457	0.872	0.745	1079	2515	−0.7	45.5	44.9	50.8
58–71	0.4227	0.4519	0.872	0.756	996	2515	−0.7	45.3	44.7	50.8
59–72	0.4142	0.4400	0.872	0.749	986	2513	−0.7	44.9	44.4	51.1
50–63	0.3985	0.4314	0.879	0.749	968	2509	−0.7	45.2	44.8	50.3
52–65	0.4274	0.4538	0.872	0.751	1063	2494	−0.9	45.8	45.3	50.3
51–64	0.4100	0.4418	0.871	0.749	1037	2460	−1.3	45.2	44.8	50.6

**Table 4:** Refinement statistics of *SEQUENCE SLIDER* hypotheses generated from helix 2 for fragments *G*+*O*, *H*+*P* and *I*+*Q* HypSeq, sequence hypothesis; RSCC, real-space correlation coefficient; mc, main-chain atoms; sc, side-chain atoms; LLG, log-likelihood gain; wMPE, weighted mean phase error; Energy, side-chain free energy as calculated with *SCWLR*4 as described in Krivov *et al.* (2009 ▸). Δcontrast is the number of standard deviations that an LLG is above the average LLG for all models involving equivalent hypotheses. wMPE* is calculated from the phases from the map from refinement rather than from coordinates.

Chains	HypSeq	*R*	*R*_free_	RSCCmc	RSCCsc	Energy	LLG	Δcontrast	wMPE (°)	wMPE* (°)	Identity (%)
*H*+*P*	77–91	0.322	0.378	0.896	0.756	938	3194	1.8	41.4	40.9	59.5
*G*+*O*	77–90	0.329	0.381	0.894	0.758	915	3172	1.0	42.4	41.9	56.1
*G*+*O*	78–91	0.332	0.381	0.895	0.758	921	3165	0.8	42.2	41.6	56.1
*H*+*P*	76–90	0.327	0.383	0.892	0.757	955	3157	0.5	42.5	41.9	56.2
*I*+*Q*	74–88	0.331	0.385	0.892	0.760	979	3157	0.5	42.0	41.3	56.1
*I*+*Q*	75–89	0.330	0.384	0.892	0.758	967	3154	0.4	42.1	41.6	55.6
*H*+*P*	74–88	0.327	0.381	0.894	0.759	1012	3153	0.4	42.8	42.2	55.2
*G*+*O*	75–88	0.337	0.383	0.895	0.760	922	3151	0.3	41.7	41.1	56.1
*G*+*O*	74–87	0.332	0.381	0.894	0.759	1001	3147	0.1	42.3	41.8	55.8
*G*+*O*	76–89	0.331	0.379	0.894	0.755	919	3146	0.1	42.4	41.7	56.1
*H*+*P*	78–92	0.329	0.386	0.892	0.758	954	3141	−0.1	42.2	41.6	56.4
*I*+*Q*	77–91	0.332	0.383	0.895	0.758	981	3134	−0.3	41.8	41.3	55.9
*I*+*Q*	76–90	0.330	0.382	0.892	0.757	963	3129	−0.5	41.8	41.1	55.9
*H*+*P*	75–89	0.328	0.378	0.894	0.755	996	3104	−1.4	42.3	41.7	55.2
*G*+*O*	79–92	0.334	0.384	0.892	0.756	916	3098	−1.6	42.2	41.6	55.6
*I*+*Q*	78–92	0.333	0.385	0.888	0.755	957	3082	−2.0	42.2	41.5	56.2

**Table 5:** Refinement statistics of *SEQUENCE SLIDER* hypotheses generated from helix 3 for fragments *G*+*O* and *I*+*Q* HypSeq, sequence hypothesis; RSCC, real-space correlation coefficient; mc, main-chain atoms; sc, side-chain atoms; LLG, log-likelihood gain; wMPE, weighted mean phase error; Energy, side-chain free energy as calculated with *SCWLR*4 as described in Krivov *et al.* (2009 ▸). Δcontrast is the number of standard deviations that an LLG is above the average LLG for all models involving equivalent hypotheses. wMPE* is calculated from the phases from the map from refinement rather than from coordinates.

Chain	HypSeq	*R*	*R*_free_	RSCCmc	RSCCsc	Energy	LLG	Δcontrast	wMPE (°)	wMPE* (°)	Identity (%)
*I*+*Q*	129–143	0.3121	0.3738	0.896	0.768	1015	3572	2.2	40.5	40.2	63.2
*G*+*O*	129–142	0.3165	0.3707	0.896	0.764	1013	3523	1.1	41.2	40.6	59.8
*I*+*Q*	138–152	0.3123	0.3744	0.895	0.766	1197	3512	0.9	40.9	40.6	59.1
*G*+*O*	128–141	0.3193	0.3724	0.896	0.761	1006	3509	0.9	41.1	40.6	59.7
*G*+*O*	127–140	0.3142	0.3730	0.892	0.761	1016	3505	0.8	41.3	40.9	60.0
*G*+*O*	132–145	0.3127	0.3714	0.895	0.762	1014	3498	0.6	41.3	40.8	59.7
*G*+*O*	136–149	0.3206	0.3703	0.895	0.764	1027	3492	0.5	41.4	40.8	59.7
*G*+*O*	130–143	0.3129	0.3750	0.894	0.759	1005	3488	0.4	41.2	40.7	59.8
*G*+*O*	135–148	0.3178	0.3722	0.893	0.758	1015	3487	0.4	41.3	40.8	59.7
*I*+*Q*	130–144	0.3193	0.3814	0.896	0.765	1094	3485	0.4	41.6	41.1	58.9
*I*+*Q*	132–146	0.3169	0.3755	0.894	0.757	1308	3485	0.4	41.6	41.1	59.5
*G*+*O*	134–147	0.3186	0.3790	0.895	0.758	1028	3481	0.3	41.5	40.9	59.7
*G*+*O*	137–150	0.3145	0.3743	0.896	0.762	1006	3475	0.2	41.2	40.6	59.7
*G*+*O*	138–151	0.3202	0.3774	0.896	0.759	1015	3463	−0.1	41.4	40.9	59.7
*G*+*O*	139–152	0.3182	0.3721	0.894	0.760	1005	3463	−0.1	41.1	40.5	59.8
*G*+*O*	131–144	0.3157	0.3734	0.895	0.761	1015	3456	−0.2	41.1	40.6	59.8
*I*+*Q*	134–148	0.3159	0.3713	0.896	0.758	1216	3450	−0.3	41.0	40.6	59.4
*I*+*Q*	135–149	0.3174	0.3720	0.897	0.760	1223	3442	−0.5	41.3	40.7	59.1
*I*+*Q*	133–147	0.3183	0.3726	0.893	0.762	1146	3439	−0.5	40.9	40.5	60.2
*I*+*Q*	127–141	0.3154	0.3760	0.892	0.759	1176	3439	−0.6	41.1	40.7	59.2
*G*+*O*	133–146	0.3167	0.3792	0.894	0.757	1011	3439	−0.6	41.2	40.8	59.7
*I*+*Q*	137–151	0.3168	0.3726	0.895	0.760	1197	3423	−0.9	41.6	41.1	59.4
*I*+*Q*	128–142	0.3187	0.3736	0.895	0.762	1422	3421	−0.9	41.6	41.1	59.2
*I*+*Q*	131–145	0.3145	0.3731	0.893	0.759	1266	3395	−1.5	41.9	41.5	59.1
*I*+*Q*	136–150	0.3186	0.3803	0.894	0.763	1217	3317	−3.0	41.4	41.1	58.6

**Table 6:** Summary of the LLG contribution of each chain in the *SEQUENCE SLIDER* cycles run on partial solutions of MltC The values correspond to the LLG variation upon omitting the particular chains, the r.m.s.d. (Å) against the final structure, the *B* factor (Å^2^) in the final structure and the number of residues in the fragments. Chains selected for sequence assignment in which a single hypothesis was distinguished are highlighted in bold.

	First *SEQUENCE SLIDER* cycle				Second *SEQUENCE SLIDER* cycle				Third *SEQUENCE SLIDER* cycle			
Chains traced	LLG	R.m.s.d.	*B*	No. of residues	LLG	R.m.s.d.	*B*	No. of residues	LLG	R.m.s.d.	*B*	No. of residues
*G*+*O*	65	0.4	49.3	14	109	0.8	52.4	28	184	0.4	52.2	28
*H*+*P*	77	0.6	43.8	14	**161**	**0.8**	**47.4**	**30**	—	—	—	—
*I*+*Q*	48	0.7	49.6	12	48	0.9	51.6	30	**178**	**0.6**	**51.1**	**30**
*L*+*M*	**96**	**0.8**	**40.2**	**28**	—	—	—	—	—	—	—	—

**Table 7:** Summary of *SEQUENCE SLIDER* hypotheses generation by chain in PDB entry 5lcy Abbreviations: #Res, number of residues present in secondary-structure element; PosHypAln, rank of correct hypothesis by score; LLG, log-likelihood gain; Δcontrast, difference of the correct hypothesis from the second best in σ. #H1, #H2 and #H3 refer to the number of hypotheses generated for the sequence-predicted helices 1, 2 and 3, respectively.

Chain	LLG	#Res	#H1	#H2	#H3	PosHypAln	LLG	Δcontrast
*A*	406	33	—	3	—	1/7	2560–2650	1.2
*B*	374	32	—	4	—	1/8	2530–2670	1.0
*C*	355	31	—	5	—	1/9	2460–2690	1.2
*D*	356	29	6	11	—	2/17	2550–2680	0.9
*E*+*F*+*G*+*H*	884	4 × 26	9	—	—	5/9	2745–3710	2.2
*I*+*J*+*K*+*L*	570	4 × 17	—	—	11	1/6	2440–2880	0.5

**Table 8:** Different numbers of cycles of *BUSTER* refinement of the *ARCIMBOLDO_SHREDDER* partial solution and their LLGs LLG, CC and wMPE are abbreviations for log-likelihood gain, correlation coefficient and weighted mean-phase error, respectively.

Cycle	LLG	CC	wMPE (°)
0	52.72	3.67	75.2
1	50.83	3.62	75.3
2	61.27	4.26	76.7
3	55.13	4.15	76.1
4	18.54	1.86	85.8
5	20.91	2.51	83.5
6	8.02	0.64	87.1
7	8.14	1.53	86.5
